# Analysis of lifestyle factors related to improving blood glucose metabolism by continuous glucose monitoring: a multicenter study in the Chikugo region

**DOI:** 10.1007/s13340-026-00897-3

**Published:** 2026-04-22

**Authors:** Hitomi Nakayama, Toshihiko Hashinaga, Kentaro Yamada, Nobuhiko Wada, Chizuko Inada, Shimpei Iwata, Ichiro Tokubuchi, Satoko Yoshinobu, Yohei Tanaka, Ayako Nagayama, Yoshie Otsuka, Nao Hasuzawa, Hiroko Haruta, Itaru Morikawa, Miyu Kinjo, Masatoshi Nomura

**Affiliations:** 1 Chikugo Municipal Hospital, 917-1 Izumi, Chikugo, Fukuoka 833-0041 Japan; 2 Chikugo Diabetes Endocrinology Clinic, 4-1 Maezu, Chikugo, Fukuoka 833-0002 Japan; 3 Asakura Medical Association Hospital, 422-1 Raiha, Asakura, Fukuoka 838-0069 Japan; 4 Kurume University Medical Center, 155-1 Kokubu, Kurume, Fukuoka 839-0863 Japan; 5 Inada Clinic, 1-23-2 Hanabatake, Kurume, Fukuoka 830-0039 Japan; 6 Yame General Hospital, 540-2 Takatsuka, Yame, Fukuoka 834-0034 Japan; 7 Omuta City Hospital, 2-19-1 Takarazaka, Omuta, Fukuoka 836-8567 Japan; 8 Kurume General Hospital, 21 Kushihara, Kurume, Fukuoka 830-0013 Japan; 9 Division of Endocrinology and Metabolism, Department of Internal Medicine, Kurume University School of Medicine, 67 Asahimachi, Kurume, Fukuoka 830-0011 Japan

**Keywords:** Continuous glucose monitoring, Dietary habit, Exercise habit, Insulin injection, Life-style, Questionnaire

## Abstract

**Background:**

Although lifestyle-improving effects of continuous glucose monitoring (CGM) have long been suggested, it remains unclear which lifestyle changes contribute to improvements in blood glucose control. This multicenter study evaluated the effect of CGM on glycosylated hemoglobin (HbA1c) and identified lifestyle factors associated with its improvement.

**Methods:**

We retrospectively investigated changes in insulin injection methods and lifestyle habits after starting CGM via a questionnaire and analyzed the associations between these changes and the degree of reduction in HbA1c over a 6-month period.

**Results:**

Wearing a CGM device reduced HbA1c by a mean of 0.83% (median 0.5%), regardless of sex or age. This reduction was smaller in individuals with type 1 diabetes and in those receiving insulin injections 3 or more times per day. Changes in insulin injection doses were not the primary cause of the reduction in HbA1c. Increased insulin doses and more frequent adjustments were not associated with lower HbA1c. After adjusting for HbA1c levels at the start of CGM use, dietary changes associated with HbA1c improvement included reduced intake of carbohydrates and foods that increase blood glucose levels and decreased meal skipping. Increases in resistance exercise and postprandial exercise were also associated with this improvement.

**Conclusions:**

The decrease in HbA1c levels after starting CGM was driven primarily by participants becoming aware of fluctuations in blood glucose levels due to diet and exercise, and making lifestyle changes to improve glycemic control.

## Introduction

Wearing a continuous glucose monitoring (CGM) device allows individuals with diabetes to observe the effects of diet and exercise on blood glucose and learn about the relationship. It has been known that CGM can change eating behavior [1, 2], and it is believed that the ability of CGM to improve blood glucose control is due not only to the optimization of treatment but also to improvements in eating habits. For exercise, it has been reported that wearing a CGM device discourages exercise, which may cause hypoglycemia in persons with type 1 diabetes [3], but promotes exercise to lower blood glucose in those with type 2 diabetes and prediabetes, strengthening exercise habits [4, 5]. However, most reports on the lifestyle-improving effects of wearing a CGM device have been narrative reviews [2], qualitative studies [6] or small-group pilot studies [7–11], and few have demonstrated with statistical significance what lifestyle habits are improved and what factors (disease type, sex, age, medication, etc.) are associated with that improvement [12–14]. Therefore, we used a questionnaire survey to clarify the changes in lifestyle habits associated with wearing a CGM device, and investigated the relationships between these changes and improvements in glycemic control in the real world.

## Methods

### Study participants

The study participants were selected from diabetic persons treated at Kurume University Hospital and affiliated hospitals in the Chikugo region in Kyushu, Japan, on the basis of the following criteria.

1. 18 years old or older and under 80 years old.
2. Persons who were receiving insulin treatment.
3. Use FreeStyle Libre or Libre 2 (Abbott Laboratories, Chicago, USA) or Dexcom G6 (Dexcom Inc. San Diego, USA) for 6 months or more.

Individuals who fell under any of the following categories were not included in this study.

1. Stage 4 or 5 diabetic nephropathy.
2. Severe autonomic neuropathy.
3. Severe liver dysfunction.
4. New York Heart Association class III or higher heart failure.
5. Hyperthyroidism.
6. Active infection.
7. Symptomatic malignant neoplasm.
8. Psychiatric disorders affecting self-care.
9. Pregnant.

## Study design

On the day of the patient’s visit, the physician researchers explained the purpose of the study, the survey items, freedom to participate, protection of personal data, and contact information via a questionnaire request form. The first question asked participants to tick the informed consent question to express their agreement as a requirement for completing the survey about behavioral changes after starting CGM. The participants who provided their consent were asked to complete the questionnaire anonymously. The questionnaire contained the following items.

1. Insulin injections.

It became easier to understand the glucose-lowering effect of insulin.

I increased the insulin dose.

I adjusted the bolus insulin dose more frequently on the basis of carbohydrate consumption.

I adjusted the bolus insulin dose more frequently on the basis of premeal glucose levels.

(2) Eating behavior.

I realized that blood glucose levels rise differently depending on the type of food.

I overate less often than before.

I started to avoid foods that increase blood glucose.

I ate staple foods less than before.

I ate carbohydrates less than before.

I ate greasy foods less than before.

I ate green and yellow vegetables more than before.

I started eating vegetables first more often.

I snacked less than before.

I skipped meals less often.

(3) Exercise.

I realized that exercise lowers my blood glucose.

I increased aerobic exercise, such as walking, compared with before.

I increased muscle-building resistance exercise more than before.

I increased exercise after meals.

I spent less time sitting*.*

The coresearchers recorded the subject’s age, sex, start date of CGM, and HbA1c value at the start of CGM and at 6 months after starting CGM on the medical data sheet. Instead of personal identifiable information such as names or hospital IDs, the physicians wrote a pseudonymized number specific to this study. The same number was written on the questionnaire. The coresearchers at each hospital recorded the survey number and hospital ID in case consent was withdrawn. The completed questionnaire and the medical data were placed in an envelope, sealed and transferred to the principal investigator. The anonymized data were reviewed by statistical analysts.

The study protocol followed the World Medical Association’s Declaration of Helsinki and was approved by the Research Ethics Committee of Kurume University School of Medicine (approval number: 23197, Jan 30, 2024).

## Statistical analysis

Data are shown as the means and SDs, or medians and interquartile ranges. The Mann–Whitney U Test was used to compare the changes in HbA1c after CGM was worn between the two groups. Pearson regression analysis was used to test for correlations between two continuous variables. Multiple regression analysis was performed with EZR, which is a graphical user interface for R.

## Results

Of the 170 individuals who agreed to participate in this study and responded to the questionnaire, 140 met all the criteria and had all the necessary data. The subjects of this study were 74 men and 66 women, aged 60.0 ± 13.3 (mean ± SD, median 61) years. Background characteristics are shown in Table 1.

**Table 1:** Background characteristics of participants

Gender (n)	Male 74, Female 66
Age (years)	60.0 ± 13.3
Type of diabetes (n)	Type 1, 69; Type 2, 64; Others, 7
Baseline HbA1c (%)	8.37 ± 1.43
Number of insulin injections per day (n)	One, 36; Two, 13; Three or more, 91
CGM device (n)	Libre 88, Libre 2 46, Dexcom G6 6
Means and SD	

**Fig. 1:**
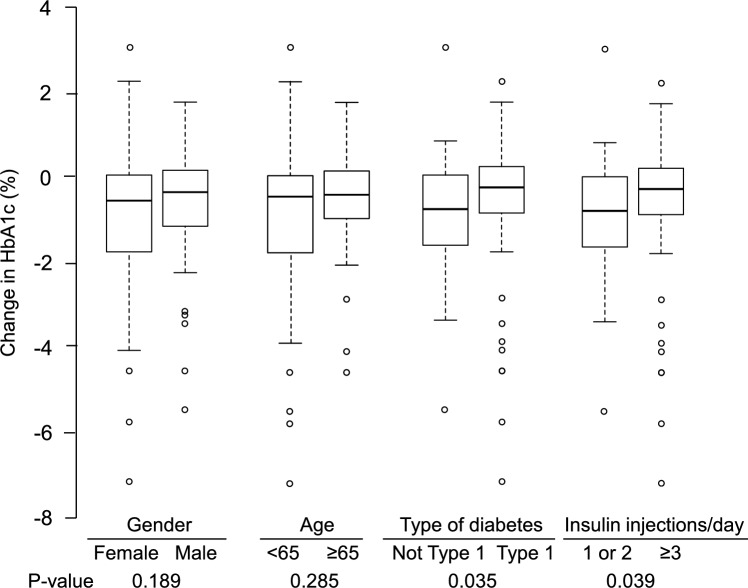
Association between clinical characteristics and changes in HbA1c. The results are shown as median and interquartile range values. *P*-values were obtained by the Mann–Whitney’s U test

Wearing a CGM device for 6 months reduced HbA1c by an average of 0.83% (median 0.5%). Eighty-eight persons used Freestyle Libre, 46 Libre2 and 6 Dexcom G6. All data were analyzed together because no significant difference was observed in the effect of starting CGM on HbA1c levels among these devices. There was no difference between men and women or between younger individuals aged < 65 years and elderly individuals aged ≥ 65 years (Fig. 1). The reduction in HbA1c was smaller in type 1 diabetes than in non-type 1 diabetes. Similarly, the effect of CGM on HbA1c was weaker in people who were injected with insulin three or more times a day.

**Fig. 2:**
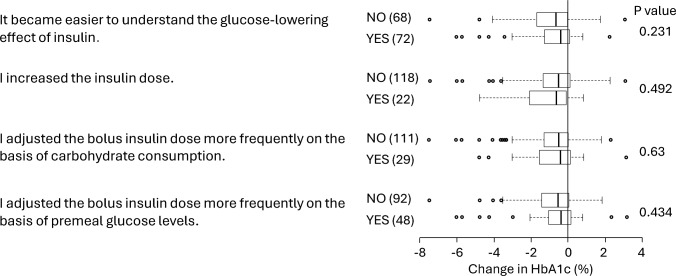
Association between alterations in insulin treatment and changes in HbA1c after starting CGM. The number of subjects is shown in brackets. The results are shown as median and interquartile ranges.* P*-values were obtained by the Mann–Whitney’s U test

We analyzed the associations of changes in HbA1c after CGM with questionnaire items. More than half of the participants answered that wearing a CGM device made it easier to understand the blood glucose-lowering effect of insulin (Fig. 2). However, this awareness was not associated with the degree of reduction in HbA1c after starting CGM. The improvement in HbA1c was not due to an increase in the insulin injection dose. An increased frequency of insulin dose adjustment on the basis of carbohydrate counting or premeal blood glucose levels did not correlate with reductions in HbA1c levels (Fig. 2).

**Fig. 3:**
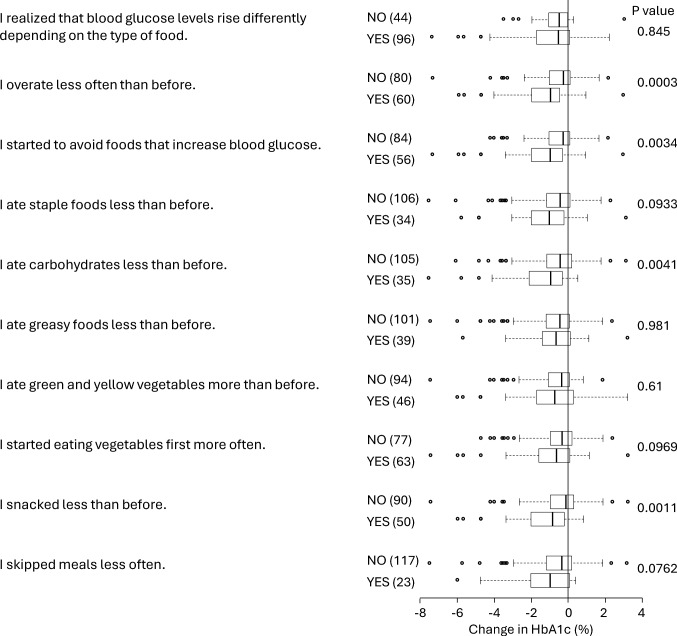
Association between alterations in eating behavior and changes in HbA1c after starting CGM. The number of subjects is shown in brackets. The results are shown as median and interquartile ranges. *P*-values were obtained by the Mann–Whitney’s U test

Approximately 70% of participants realized that blood glucose increases depend on the type of food, although this awareness itself was not related to a decrease in HbA1c (Fig. 3). Both reducing overeating and avoiding foods that tend to increase blood glucose were significantly associated with a reduction in HbA1c. Less carbohydrate intake was also associated with greater decreases in HbA1c, whereas greasy food restriction was not. Participants who reduced their level of snacking had greater reductions in HbA1c after CGM was worn. However, a reduced intake of staple foods or an increased intake of vegetables was not associated with improved glycemic control. People who reduced meal skipping tended to have a larger reduction in HbA1c, but this difference was not statistically significant.

**Fig. 4:**
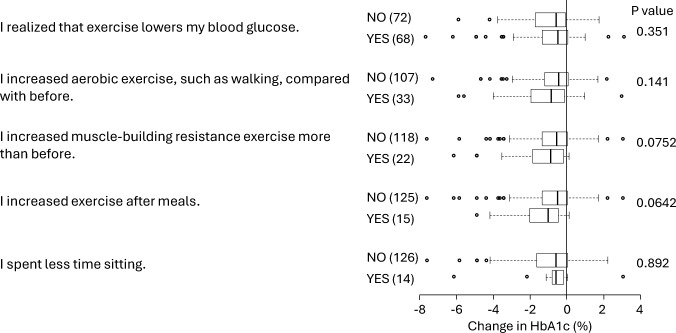
Association between alterations in exercise habits and changes in HbA1c after starting CGM. The number of subjects is shown in brackets. The results are shown as median and interquartile ranges. *P*-values were obtained by the Mann–Whitney’s U test

Although approximately half of the participants reported a decrease in blood glucose levels after exercise, the percentage of people who actually increased their exercise was low (Fig. 4). Neither an increase in aerobic nor resistance exercise was significantly associated with improvements in HbA1c. A reduction in sitting time was not associated with an improvement in HbA1c.

**Fig. 5:**
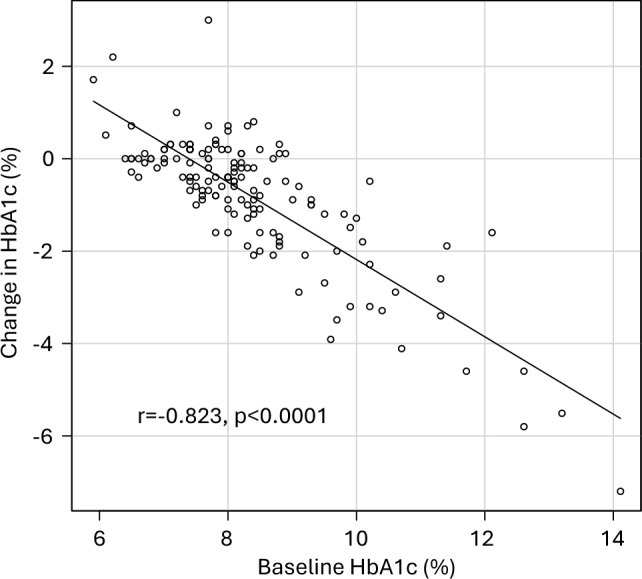
Correlation between changes in HbA1c over a 6-month period and baseline HbA1c at the start of CGM wearing

The reduction in HbA1c over the 6 months after CGM use was strongly correlated with the HbA1c value at the start of CGM (Fig. 5). Therefore, we performed multiple regression analysis to eliminate the influence of baseline HbA1c values (Table 2). Type 1 diabetes and ≥ 3 insulin injections per day were associated with smaller reductions in HbA1c, independent of baseline HbA1c levels. No item related to insulin injections was associated with a reduction in HbA1c, even when adjusting for baseline HbA1c values. There was a significant correlation between awareness that blood glucose increases differently depending on the type of food and the degree of reduction in HbA1c. It was confirmed that reducing carbohydrates and foods that increase blood glucose levels was associated with improved glycemic control. Furthermore, there was a significant correlation between a reduction in skipping meals and improvements in HbA1c. With respect to exercise habits, an increase in resistance exercise, but not aerobic exercise, was significantly correlated with an improvement in HbA1c. However, increased postprandial exercise was associated with this phenomenon (Table 2).

**Table 2:** Correlation between variables and changes in HbA1c after adjusting for basal HbA1c values

		β (95% CI)	SE	t-value	*p*-value
*Clinical characteristics*					
	Male gender	0.0952 (− 0.1847–0.375)	0.1415	0.6725	0.5024
	Age ≧65	− 0.014 (− 0.2983–0.2703)	0.1438	− 0.0972	0.9227
	Type 1 diabetes	0.2804 (0.0059–0.5549)	0.1388	2.0197	0.0454*
	Three or more insulin injections per day	0.3295 (0.0408–0.6182)	0.146	2.2568	0.0256*
*Insulin injections*					
	It became easier to understand the glucose-lowering effect of insulin	0.0291 (− 0.0669–0.1251)	0.0485	0.5993	0.5500
	I increased the insulin dose	0.1075 (− 0.2772–0.4921)	0.1945	0.5524	0.5816
	I adjusted the bolus insulin dose more frequently on the basis of carbohydrate consumption	0.2058 (− 0.136–0.5476)	0.1729	1.1904	0.2359
	I adjusted the bolus insulin dose more frequently on the basis of premeal glucose levels	0.1931 (− 0.0984–0.4847)	0.1474	1.3098	0.1924
*Eating behavior*					
	I realized that blood glucose levels rise differently depending on the type of food	− 0.304 (− 0.5995– − 0.0085)	0.1494	− 2.0342	0.0439*
	I overate less often than before	− 0.2332 (− 0.5178–0.0513)	0.1439	− 1.6206	0.1074
	I started to avoid foods that increase blood glucose	-0.3056 (− 0.5894– − 0.0218)	0.1435	− 2.1296	0.035*
	I ate staple foods less than before	− 0.0194 (− 0.3469–0.3081)	0.1656	− 0.1172	0.9069
	I ate carbohydrates less than before	0.5445 (− 0.8547– − 0.2342)	0.1569	− 3.4702	0.0007*
	I ate greasy foods less than before	0.1398 (− 0.1699–0.4494)	0.1566	0.8926	0.3736
	I ate green and yellow vegetables more than before	− 0.0545 (− 0.3516–0.2426)	0.1502	− 0.3627	0.7174
	I started eating vegetables first more often	− 0.1866 (− 0.4654–0.0923)	0.141	− 1.3231	0.188
	I snacked less than before	− 0.2464 (− 0.5413–0.0486)	0.1491	− 1.6519	0.1009
	I skipped meals less often	− 0.6725 (− 1.0307–-0.3142)	0.1812	− 3.7117	0.0003*
*Exercise*					
	I realized that exercise lowers my blood glucose	− 0.0268 (− 0.3055–0.2518)	− 0.1905	− 0.1905	0.8492
	I increased aerobic exercise, such as walking, compared with before	− 0.2862 (− 0.611–0.0385)	− 1.7432	− 1.7432	0.0835
	I increased muscle-building resistance exercise more than before	− 0.4203 (− 0.7975– − 0.0431)	− 2.2035	− 2.2035	0.0292*
	I increased exercise after meals	− 0.5402 (− 0.9812– − 0.0992)	− 2.4223	− 2.4223	0.0167*
	I spent less time sitting	− 0.032 (− 0.4968–0.4328)	− 0.1362	− 0.1362	0.8919

Baseline HbA1c was used as an independent variable. β, standardized coefficient; SE, standard error, *p < 0.05

## Discussion

In this study, we hypothesized that the decrease in HbA1c levels after starting CGM was caused mainly by participants being aware of blood glucose fluctuations caused by diet and exercise and changing their lifestyle to improve blood glucose control. Thus, we conducted a questionnaire survey to clarify what lifestyle changes occurred after wearing a CGM device and to examine which changes were associated with improved blood glucose control.

The HbA1c level significantly decreased 6 months after wearing CGM (Fig. 1). Previous reports on the usefulness of CGM in elderly people have shown conflicting results [15–18]. In this study, no sex or age differences were found in the improvement of HbA1c levels by wearing a CGM device, suggesting that CGM is useful for elderly persons as long as it is used appropriately. The reason why the decrease in HbA1c was greater in type 2 diabetes than in type 1 diabetes is probably because lifestyle factors play a greater role in type 2 diabetes. The same reason may be applicable to the weaker effect in those who had received 3 or more insulin injections. In cases of type 1 diabetes or intensive insulin therapy, the main benefit of CGM is likely not to lower average blood glucose levels but to stabilize blood glucose fluctuations and prevent hypoglycemia. Although not examined in this study, CGM was also effective in improving lifestyle habits in diabetic persons who do not use insulin, and even in prediabetic persons and obese subjects with normal glucose tolerance [19–21].

Wearing a CGM device made the rise in blood glucose after a meal visible, and 68.6% of the participants realized that the increase in blood glucose differed depending on the type of food (Fig. 3). People who realize this will be able to avoid foods that are likely to increase blood glucose levels. In fact, participants who did so experienced a greater reduction in HbA1c. Therefore, making people aware that postprandial blood glucose levels vary depending on food may be crucial in improving blood glucose control after wearing a CGM device. The association between the reduction in carbohydrate intake after starting CGM and HbA1c improvement may also be due to the awareness that carbohydrate intake causes a large increase in blood glucose. Even without knowledge about carb counting, avoiding foods that are likely to cause blood glucose increases would ultimately have the same effect (Fig. 3).

Interestingly, a reduction in skipping meals was strongly associated with a decrease in HbA1c (Table 2). Skipping meals, especially breakfast, may result in increased total energy intake and elevated blood glucose levels because meta-analyses have shown that skipping breakfast is associated with an increased risk of overweight/obesity [22] and type 2 diabetes [23]. The use of CGM devices likely made participants realize that skipping meals worsened their blood glucose fluctuations, contributing to improved blood glucose control.

The association between increased aerobic exercise and improved HbA1c did not reach statistical significance even after adjusting for basal HbA1c. However, increased resistance exercise and increased postprandial exercise were both associated with improvements in HbA1c. It is well known that aerobic exercise reduces HbA1c, but it has been reported that resistance exercise has similar effects on improving blood glucose [24, 25]. The reason why postprandial exercise was effective is likely because CGM made participants aware of the acute effect of exercise in lowering blood glucose levels, prompting exercise after meal to suppress postprandial hyperglycemia.

On the other hand, awareness of the blood glucose lowering effect of exercise through the use of a CGM device results in the prevention of exercise-induced hypoglycemia [26]. CGM devices teach participants about the blood glucose lowering effect of exercise and discourage them from engaging in exercise that may cause hypoglycemia [27]. Furthermore, participants may have consumed carbohydrates to prevent hypoglycemia when performing aerobic exercise. These findings may explain why only resistance exercise and exercise after meals, which are less likely to cause hypoglycemia, were associated with a decrease in HbA1c*.*

## Limitations

This study has several limitations. First, lifestyle changes were analyzed via a questionnaire, and no dietary interview to evaluate dietary intake or objective physical activity measurement via an accelerometer was performed. Second, real-time CGM and intermittently scanned CGM were analyzed together without distinction because few people wore the real-time CGM device, and the Freestyle Libre 2 can be used as both a real-time and an intermittently scanned CGM. Third, in individuals using intermittently scanned CGM, more frequent scanning has been associated with higher time in range [28]. However, scanning frequency was not assessed in this study. Fourth, improvement in glycemic control was evaluated only by HbA1c values. Although HbA1c values allowed us to compare glycemic control 6 months after the start of CGM use with that immediately before CGM use, we were unable to evaluate diurnal glycemic variation or hypoglycemic risk.

## Conclusions

CGM allows diabetic adults, including elderly individuals, to understand the effects of diet and exercise on blood glucose levels, motivating them to improve their lifestyle behaviors to achieve better blood glucose control. The results of this study are expected to be useful in establishing instruction methods to increase the effectiveness of CGM in improving blood glucose control.
